# Supplementary material for: Dynamics of the charge transfer to solvent process in aqueous iodide
Source: Nat Commun. 2024 Mar 21;15:2544. doi: 10.1038/s41467-024-46772-0 (PMC11258362; doi:10.1038/s41467-024-46772-0)
Supplement: Supplementary file 1 — Supplementary Information [file 41467_2024_46772_MOESM1_ESM.pdf]

Supplementary Information for Dynamics of the Charge Transfer to Solvent Process in Aqueous Iodide

Jinggang Lan,[1,2,3,*] Majed Chergui,[4,5] and Alfredo Pasquarello[1]

[1]*Chaire de Simulation à l'Echelle Atomique (CSEA),*

Ecole Polytechnique Fédérale de Lausanne (EPFL), CH-1015 Lausanne, Switzerland

[2]*Department of Chemistry, New York University, New York, NY, 10003, USA*

[3]*Simons Center for Computational Physical Chemistry at*

New York University, New York, New York 10003, USA

[4]*Lausanne Centre for Ultrafast Science (LACUS), ISIC,*

Ecole Polytechnique Fédérale de Lausanne (EPFL), CH-1015 Lausanne, Switzerland

[5]*Elettra - Sincrotrone Trieste S.C.p.A.S.S.14 Km.163,*

5 in Area Science Park I - 34149 Trieste, Italy

[*] jinggang.lan@nyu.edu

Contents

1. Time dependent density functional theory 3

2. Benchmark: S_1 vs T_1 4

3. Machine Learning 6

4. Free energy calculations 8

5. Dynamics 10

6. Nuclear quantum effects correction on energy level 13

7. Smoluchowski diffusion equation 13

8. Center and gyration radius of a localized charge density 14

Supplementary Note 1. Time dependent density functional theory

We carry out time dependent density functional theory (TDDFT) calculations for configurations of aqueous iodide simulations using a QM/MM protocol as implemented in the ORCA code [1]. For each adsorption spectrum, we use 50 configurations homogeneously distributed in time between 0 and 100 fs. The QM region comprises 70 water molecules closest to the center of the iodine, corresponding to the cluster with the largest radius fully contained in the simulation cells. The MM region is described through a point charge model and constructed on the basis of 560 surrounding water molecules in the periodic supercells. The rest of the environment is described by the conductor-like polarizable continuum model [2] with the experimental dielectric constant of water. In the TDDFT calculations, we use the PBEh(0.40) functional with the ma-def2-TZVP basis set [3] and consider the first five excited states.

The absorption spectra presented in the accompanying figure exhibit distinct characteristics at various time delays. In the left figure, we analyze a single CTTS trajectory and calculate the absorption spectra from 0-100 fs using a time step of 2 fs. In the right figure, we average the spectra over three time intervals: 0-50 fs, 50-100 fs, and 500-1000 fs. Specifically, for the 500-1000 fs, we select 50 snapshots evenly distributed across the time interval.

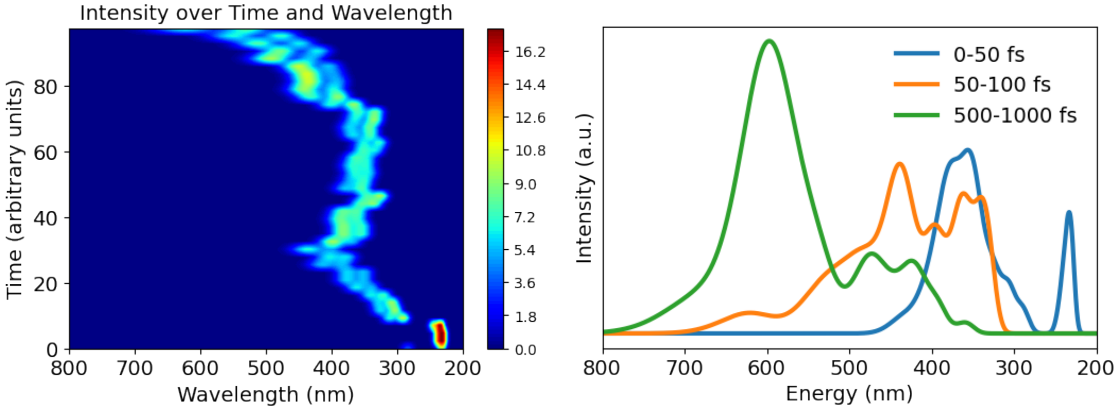

Supplementary Figure 1: (Left) Time resolved absorption spectrum obtained with a TDDFT calculation. (Right) Absorption spectrum at different time windows, 0-50 fs, 50-100 fs and 500-1000 fs.

In the experiment, the signatures derived from transient absorption spectra do not di-

rectly reflect the CTTS dynamics, as the signals from solvated electrons predominantly overshadow the entire spectrum. Therefore, we compare our calculated spectra with experimental fluorescence spectra. It is important to note that our comparison is qualitative due to the Stokes shift between absorption and emission.[4] Upon excitation of the iodide, the experimental fluorescence spectra presents a broad intensity distribution ranging from 280 to 650 nm, with a pronounced intensity peak near 340 nm. Correspondingly, our simulations in the time window 0-50 fs reveal a spectrum extending from 200 to 450 nm, with notable peaks at approximately 230 nm and 340 nm. In the time window 50-100 fs, the simulated spectrum undergoes a red shift, culminating in a significant peak near 400 nm. As the dynamics progress beyond 500 fs, both experimental and simulated spectra show a convergence towards a narrower intensity distribution and the formation of a single peak around 600 nm. These results offer valuable insights for future experiments, particularly highlighting the unique spectral features of the iodine-water complex formation as a function of time.

Supplementary Note 2. Benchmark: S_1 vs T_1

The p shell of the aqueous iodide anion is fully occupied showing the electron configuration $5p^6$. The singlet state of aqueous iodide is characterized by a p orbital as the highest occupied molecular orbital (HOMO). When the aqueous iodide anion is excited by the laser, one electron from the singlet HOMO state is excited to the lowest-unoccupied molecular orbital (LUMO). This excited state corresponds to a singlet state (S_1) when the spin configuration is preserved and becomes a triplet state (T_1) upon spin flip. The former state can be described using the TDDFT method [5], while the latter one can be obtained with unrestricted Kohn-Sham (UKS) calculations. Both S_1 and T_1 calculations describe the same electronic configuration when the excited electron separates from the aqueous iodine ($5p^5$). Upon separation, the excited electron will then form a hydrated electron either with an unflipped spin (S_1) or with a flipped spin (T_1). However, the computational cost of TDDFT is several times more expensive than UKS, due to the calculations of transitions between occupied and excited states. To benchmark the feasibility of excited-state calculation using the T_1 electronic configuration, we use reference configurations taken from a singlet excited-state molecular dynamics trajectory as obtained with TDDFT [5]. Energies and forces are recalculated using the T_1 electronic structure for such configurations. The cross-validation of

energies and forces is shown in Fig. S2. It is evident that energies and forces as obtained with T_1 and S_1 calculations are in good agreement. This agreement stems from the large band gap (> 5 eV) between the two singly occupied molecular orbitals (SOMO-1 and SOMO), resulting in minimal mixing between the two states. Hence, the structure and the dynamics pertaining to the iodine radical and the hydrated electron can equivalently be obtained with either approach. To further illustrate this equivalence in the charge transfer from solute to solvent process, we compare in Fig. S3 the electron and hole densities as obtained from the natural bond-orbital analysis in a TDDFT S_1 calculation with the spin density obtained from an unrestricted Kohn-Sham T_1 calculation for the same structural configuration.

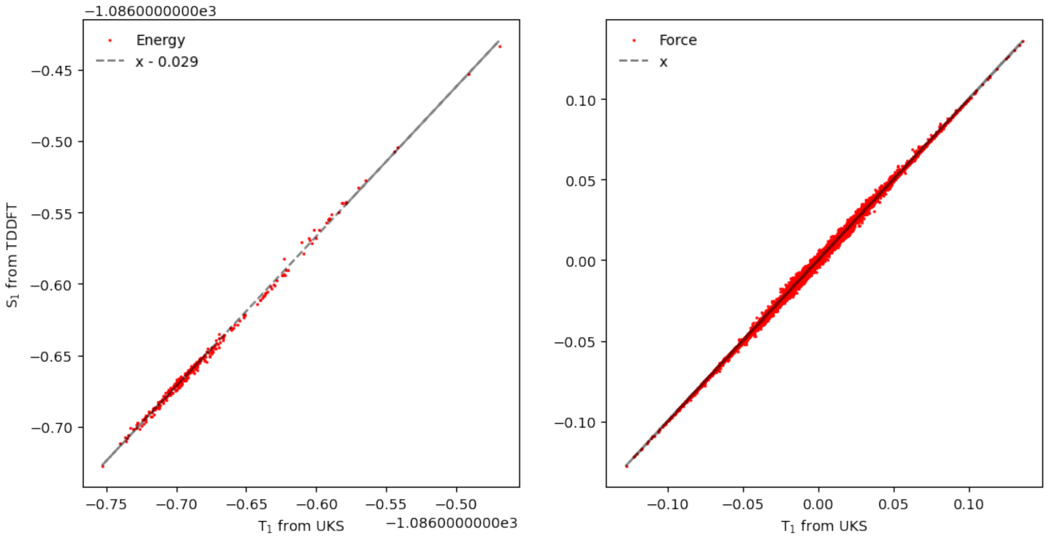

Supplementary Figure 2: Comparison between energies and forces obtained with TDDFT S_1 and unrestricted Kohn-Sham T_1 calculations.

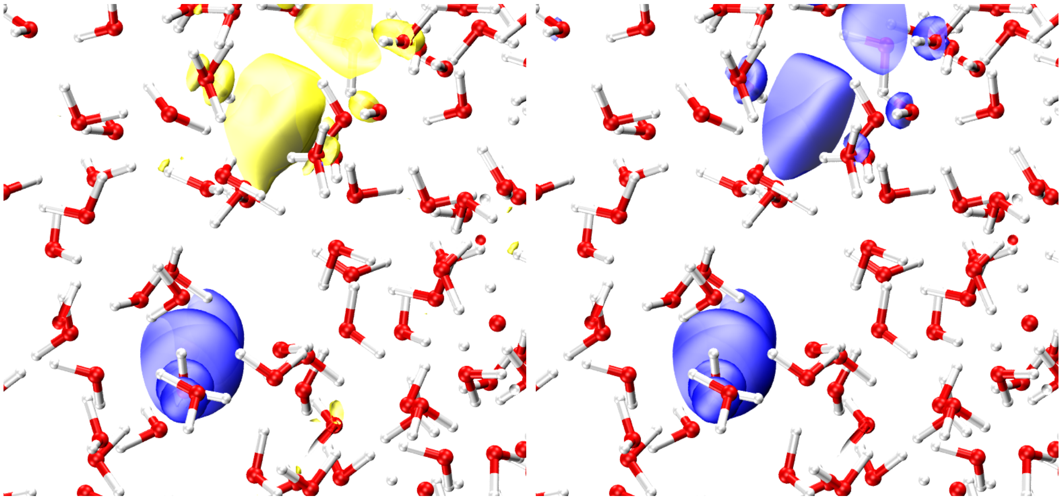

Supplementary Figure 3: Left: electron (yellow) and hole (blue) density as obtained from the natural bond-orbital analysis in a TDDFT S_1 calculation. Right: spin density as obtained from an unrestricted Kohn-Sham T_1 calculation for the same structural configuration.

Supplementary Note 3. Machine Learning

The training set is of great importance to construct an accurate machine learning potential (MLP). The initial data sets focus on the nuclei from a classical Newtonian perspective. The initial data sets are generated by hybrid functional molecular dynamics employing a timestep of 0.5 fs. The initial conditions for the trajectories of the CTTS dynamics are prepared by equilibrating aqueous iodide in a NVT ensemble at room temperature. To control the temperature, we use a thermostat ensuring canonical sampling through velocity rescaling (CSVR) [6] for the dynamics of the classical nuclei. To obtain comprehensive data sets that cover the phase spaces of quantum nuclei, we perform a thermostated ring-polymer molecular dynamics with 32 beads [7]. These simulations rely on machine learning potentials trained with the initial classical data sets and serve the purpose of exploring the phase space of the quantum nuclei.

We choose a few hundred configurations and recalculate the energies and forces at the hybrid functional level. Building upon the classical and quantum data sets, we then perform molecular dynamics simulations to explore a broader phase space. In doing so, we identify representative configurations based on their atomic fingerprints using CurSel [8] and re-assess

the energies and forces associated with these configurations. The details of the data sets are presented in Table 1, while the specific computational details related to the molecular dynamics are provided in the next section.

Supplementary Table 1: List of datasets used to train the machine learning potential for aqueous iodide, aqueous iodine, and the CTTS dynamics.

Method	I^-_{aq}	$I^{\cdot}_{aq}$	CTTS
AIMD	9176	5953	5484
PIMD	321	320	550
CurSel	328	475	210

The high-dimensional neural network potential proposed by Behler and Parrinello is sufficiently flexible to describe complex aqueous ions [9]. This method estimates the total energy E via a sum of atomic energies E_n based on a set of atom-centered many-body symmetry functions G_n. Atomic environments defined by cutoff radii of 12.0 bohr are used for hydrogen and oxygen atoms. We used 50 symmetry functions for hydrogen, 50 functions for oxygen, and 42 functions for iodine. The neural networks consist of two hidden layers with 20 nodes. All related data files including the MLPs, the data sets, the input files, and the molecular dynamics trajectories have been uploaded to the Materials Cloud repository (https://www.materialscloud.org/).

Detailed comparisons between energies obtained with the MLPs and with the hybrid functional are shown in Fig. S4. In the case of aqueous iodide, we obtain a root-mean-square error (RMSE) of 0.29 meV per atom for the energies and a RMSE of 51 meV/Å for the forces. In the case of aqueous iodine, we find a RMSE of 0.28 meV per atom for the energies and a RMSE of 51 meV/Å for the forces. For the CTTS dynamics, we find a RMSE of 0.59 meV per atom for the energies and a RMSE of 54 meV/Å for the forces.

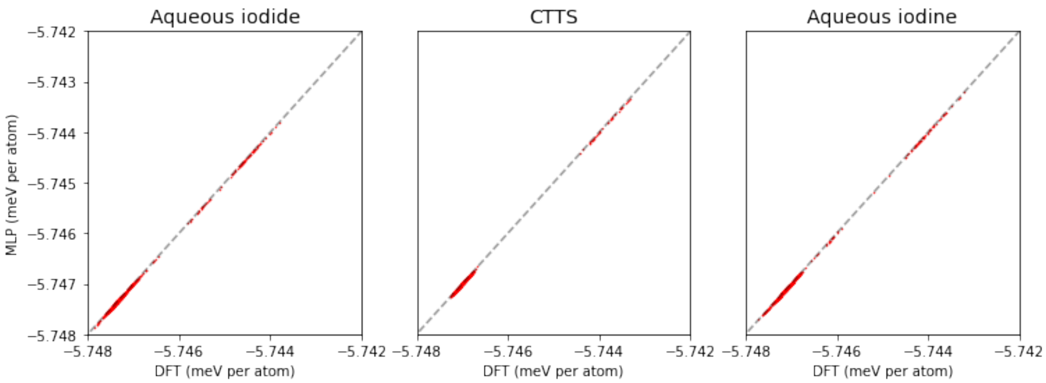

Supplementary Figure 4: Comparison between energies obtained with the hybrid functional and the machine learning potential.

Supplementary Note 4. Free energy calculations

To determine the free energy as a function of the distance between the electron and the iodine, we conduct constrained molecular dynamics simulations in which various values for the electron-iodine distance are kept fixed. The center of the electron density is approximated by the center of mass (COM) of the oxygen atoms from the water molecules that are surrounding the electron. Hence, we impose constraints on the distance between iodine and the COM. As shown in Fig. S5, this collective variable is found to agree well on average with the distance between the iodine and the gyration center of the electron density.

As illustrated in Figure S6, the time-averaged forces exerted on the collective variable, $F(x)$, are integrated over a range of distances to calculate the free energy difference using thermodynamic integration [10]:

$$\Delta A = -\int_a^b F(x)dx \quad \text{(Supplementary 1)}$$

The collective variable is assigned an initial value a of 4.0 Å and a final value b of 8.0 Å. Simulations are conducted with intervals of 0.5 Å, allowing us to explore several iodine-electron distances. A period of 1 ps is carried out at each distance to achieve system equilibration, followed by 2-ps molecular dynamics trajectory for the calculation of the time-averaged forces.

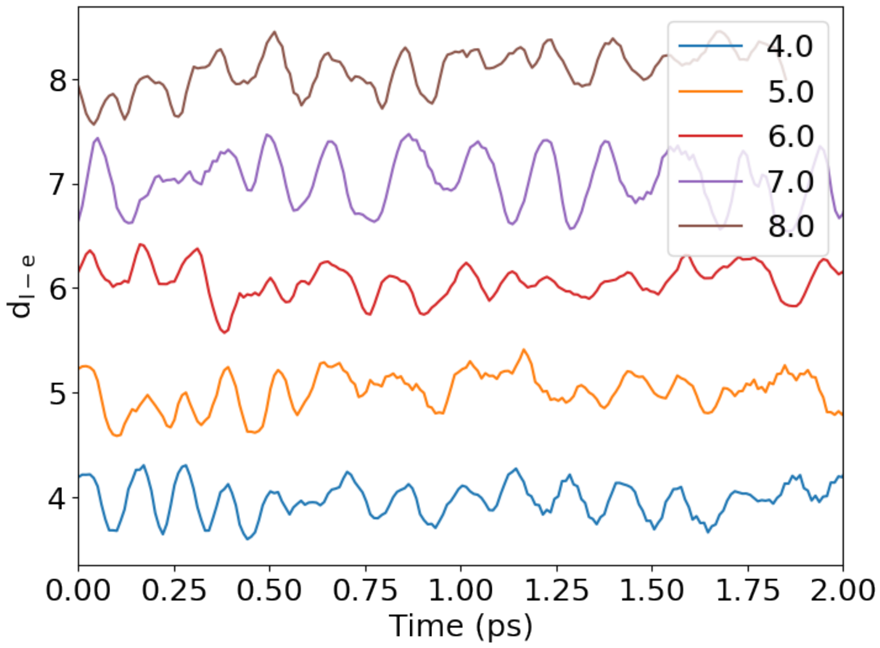

Supplementary Figure 5: Actual iodine-electron distance as a function of time as obtained from various constrained molecular dynamics in which the iodine-COM distances has been fixed (see legend).

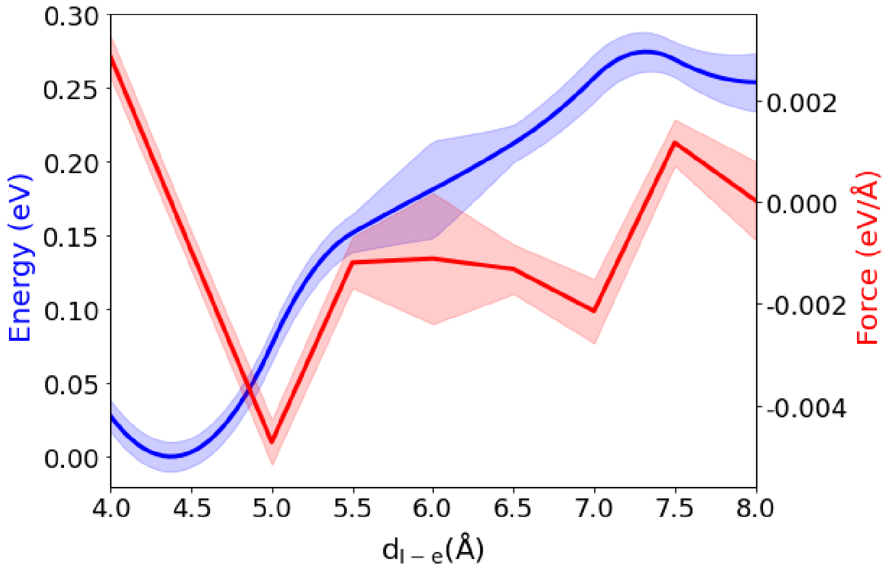

Supplementary Figure 6: Free energy as a function of iodine-electron distance (blue) and the time-averaged forces acting on the iodine-electron distance (red).

Supplementary Note 5. Dynamics

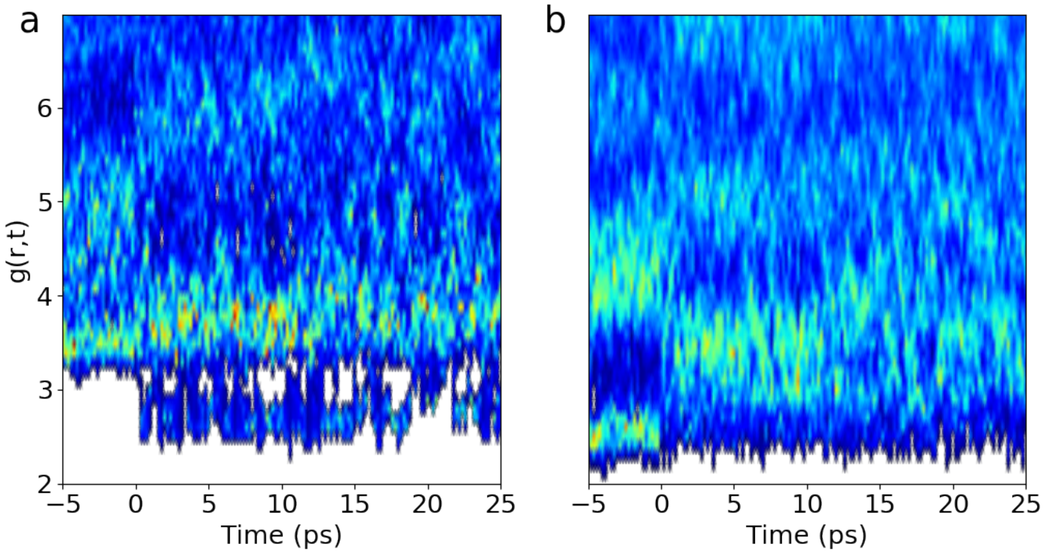

Supplementary Figure 7: Time-dependent radial distribution function for (a) I-O and (b) I-H. The structures are taken from the aqueous iodide simulation before the time origin, and from the excited-state simulation after the time origin.

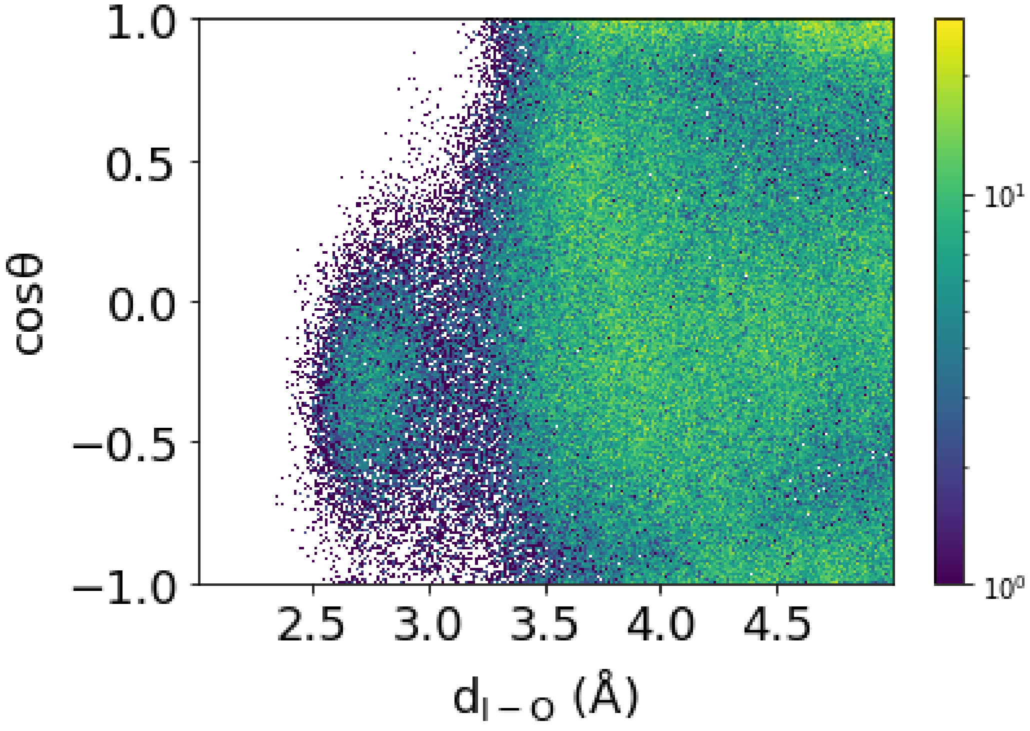

Supplementary Figure 8: Distribution of the cosine of the angle between the water dipole and the oxygen-iodine vector for the full CTTS dynamics lasting 25 ps. Positive values indicate that water dipole points towards the iodine atom, while it points outwards for negative values. As the color changes from blue to yellow, the weight of the $\cos\theta$ distribution increases.

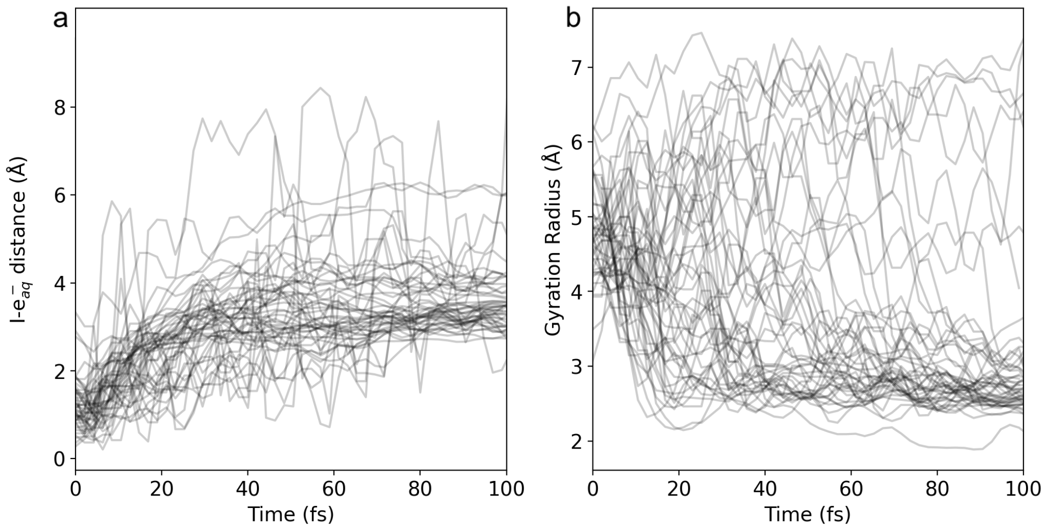

Supplementary Figure 9: (a) I-e^-_{aq} distance and (b) gyration radius as a function of time for 50 trajectories of charge-transfer-to-solvent dynamics

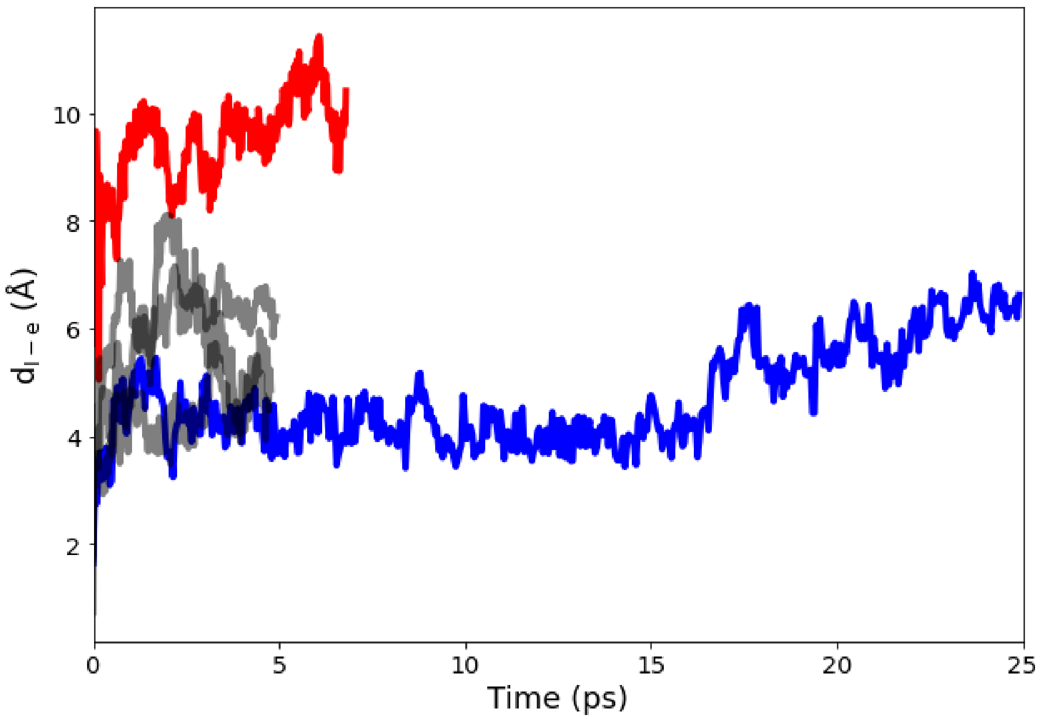

Supplementary Figure 10: I-e^-_{aq} distance as a function of time for five trajectories, among which the cases where the hydrated electron is formed in the vicinity of the iodine (blue curve) and far from the iodine (red curve) are highlighted

Supplementary Note 6. Nuclear quantum effects correction on energy level

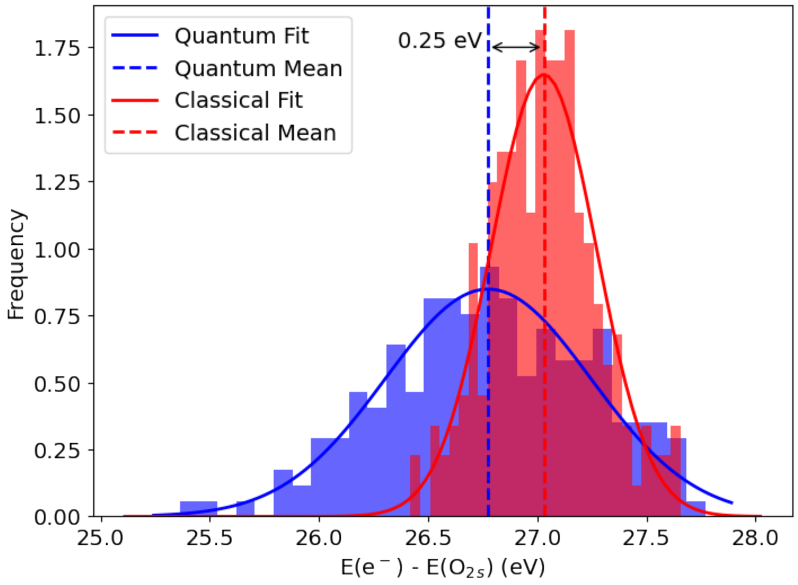

Supplementary Figure 11: Kohn-Sham orbital energy difference between e^- and O_{2s} as derived from classical and quantum dynamics

We have compared the energy levels of e^- between our classical and quantum molecular dynamics simulations. The Kohn-Sham orbital level in the quantum molecular dynamics is approximately 0.25 eV lower than that in classical molecular dynamics. In Fig. S11, we present the Kohn-Sham orbital energy difference between e^- and O_{2s} as derived from classical and quantum molecular dynamics. Given that the O_{2s} state is a deep state with a constant energy level with respect to the vacuum, we align the e^- level to the O_{2s} level [11]. Consequently, the difference in the absolute energy level of e^- between classical and quantum dynamics is about 0.25 eV.

Supplementary Note 7. Smoluchowski diffusion equation

The Smoluchowski diffusion equation has been used to analyze the free energy based on experimental measurements [12–14]. This equation is given by:

$$\frac{\partial \Psi(r,t)}{\partial t} = D' r^2 e^{-V(r)} \frac{\partial}{\partial r} r^{-1} e^{V(r)} \Psi(r,t) \qquad \text{(Supplementary 2)}$$

Here, $\Psi(r,t)$ represents the net population of hydrated electrons per unit volume, which can be calculated based on the photoelectron signal intensity. The distance of an electron from an iodine atom is denoted r, and $D^{'}$ is the diffusion coefficient.

Supplementary Note 8. Center and gyration radius of a localized charge density

The excited electron is characterized by the density of the highest occupied molecular orbital, while the hole distribution at the iodine radical is characterized by the density of lowest unoccupied molecular orbital. The center of the excited electron is calculated from the second moment tensor. Since the position operator $\mathbf{r}$ is not well defined in simulation cells subject to periodic boundary conditions, the gyration radius is not defined either. However, the simulation cell is large enough to achieve localized charge density distributions. The Gaussian cube files used to store the charge density distributions have been centered in the supercell to ensure that the charge density vanishes at the supercell boundaries.

The center of a given charge density $\rho(\mathbf{r})$ is then given as follows:

$$\mathbf{r}_c = \int \rho(\mathbf{r})\mathbf{r}d\mathbf{r}. \qquad \text{(Supplementary 3)}$$

The density of the excited electron is obtained from the square of the highest occupied molecular orbital (HOMO):

$$\rho_{\mathrm{e}}(\mathbf{r}) = |\psi_{\mathrm{HOMO}}(\mathbf{r})|^2. \qquad \text{(Supplementary 4)}$$

Similarly, one finds the hole density at the iodine radical from the square of the lowest unoccupied molecular orbital (LUMO):

$$\rho_{\mathrm{I}}(\mathbf{r}) = |\psi_{\mathrm{LUMO}}(\mathbf{r})|^2. \qquad \text{(Supplementary 5)}$$

The center of a given localized charge density ρ is then obtained by the sum over the real-space grid points:

$$\mathbf{r}_c = \sum_{i=1,2..N}^{N} \rho(\mathbf{r})\mathbf{r}, \qquad \text{(Supplementary 6)}$$

The associated second moment tensor is defined as:

$$\mathbf{S} = \int (\mathbf{r} - \mathbf{r}_c)(\mathbf{r} - \mathbf{r}_c)\rho_e(\mathbf{r})d\mathbf{r} \qquad \text{(Supplementary 7)}$$

Then, the gyration radius is calculated as:

$$r_{\text{gyr}} = \sqrt{\lambda_1^2 + \lambda_2^2 + \lambda_3^2}, \quad \text{(Supplementary 8)}$$

where λ_1, λ_2, λ_3 are the eigenvalues of $\mathbf{S}$.

Supplementary References

[1] F. Neese, Wiley Interdiscip. Rev. Comput. Mol. Sci. **2**, 73 (2012).

[2] V. Barone and M. Cossi, J. Phys. Chem. A **102**, 1995 (1998).

[3] J. Zheng, X. Xu, and D. G. Truhlar, Theor. Chem. Acc. **128**, 295 (2011).

[4] F. Messina, O. Bräm, A. Cannizzo, and M. Chergui, Nat. Commun. **4**, 2119 (2013).

[5] A.-S. Hehn, B. Sertcan, F. Belleflamme, S. K. Chulkov, M. B. Watkins, and J. Hutter, J. Chem. Theory Comput. **18**, 4186 (2022).

[6] G. Bussi, D. Donadio, and M. Parrinello, J. Chem. Phys **126**, 014101 (2007).

[7] M. Rossi, M. Ceriotti, and D. E. Manolopoulos, J. Chem. Phys. **140**, 234116 (2014).

[8] G. Imbalzano, A. Anelli, D. Giofré, S. Klees, J. Behler, and M. Ceriotti, J. Chem. Phys **148**, 241730 (2018).

[9] J. Behler and M. Parrinello, Phys. Rev. Lett. **98**, 146401 (2007).

[10] M. Sprik and G. Ciccotti, J. Chem. Phys. **109**, 7737 (1998).

[11] W. Chen, F. Ambrosio, G. Miceli, and A. Pasquarello, Phys. Rev. Lett. **117**, 186401 (2016).

[12] V. H. Vilchiz, X. Chen, J. A. Kloepfer, and S. E. Bradforth, Rad. Phys. Chem. **72**, 159 (2005).

[13] H. Iglev, A. Trifonov, A. Thaller, I. Buchvarov, T. Fiebig, and A. Laubereau, Chem. Phys. Lett. **403**, 198 (2005).

[14] Y.-i. Yamamoto, Y.-I. Suzuki, and T. Suzuki, J. Phys. Chem. Lett. **14**, 1052 (2023).
