# Supplementary material for: Dynamics of the charge transfer to solvent process in aqueous iodide
Source: Nat Commun. 2024 Mar 21;15:2544. doi: 10.1038/s41467-024-46772-0 (PMC11258362; doi:10.1038/s41467-024-46772-0)
Supplement: Supplementary file 3 — Description of Additional Supplementary Files [file 41467_2024_46772_MOESM3_ESM.pdf]

Description of Additional Supplementary Files:

Supplementary Movie 1: Ab initio molecular dynamics simulation illustrating chargetransfer-to-solvent dynamics with the formation of the hydrated electron in proximity to iodine, as indicated by the blue curve in Fig. 2g. Color code: the red spheres represent oxygen atoms, the white spheres represent hydrogen atoms, and the blue spheres represent spin density.

Supplementary Movie 2: Ab initio molecular dynamics simulation illustrating chargetransfer-to-solvent dynamics with the direct transition of aqueous iodine and the hydrated electron to a separated state, as indicated by the red curve in Fig. 2g. Color code: the red spheres represent oxygen atoms, the white spheres represent hydrogen atoms, and the blue spheres represent spin density.
