# Supplementary figures and images for: Dynamics of the charge transfer to solvent process in aqueous iodide
Source: Nat Commun. 2024 Mar 21;15:2544. doi: 10.1038/s41467-024-46772-0 (PMC11258362; doi:10.1038/s41467-024-46772-0)

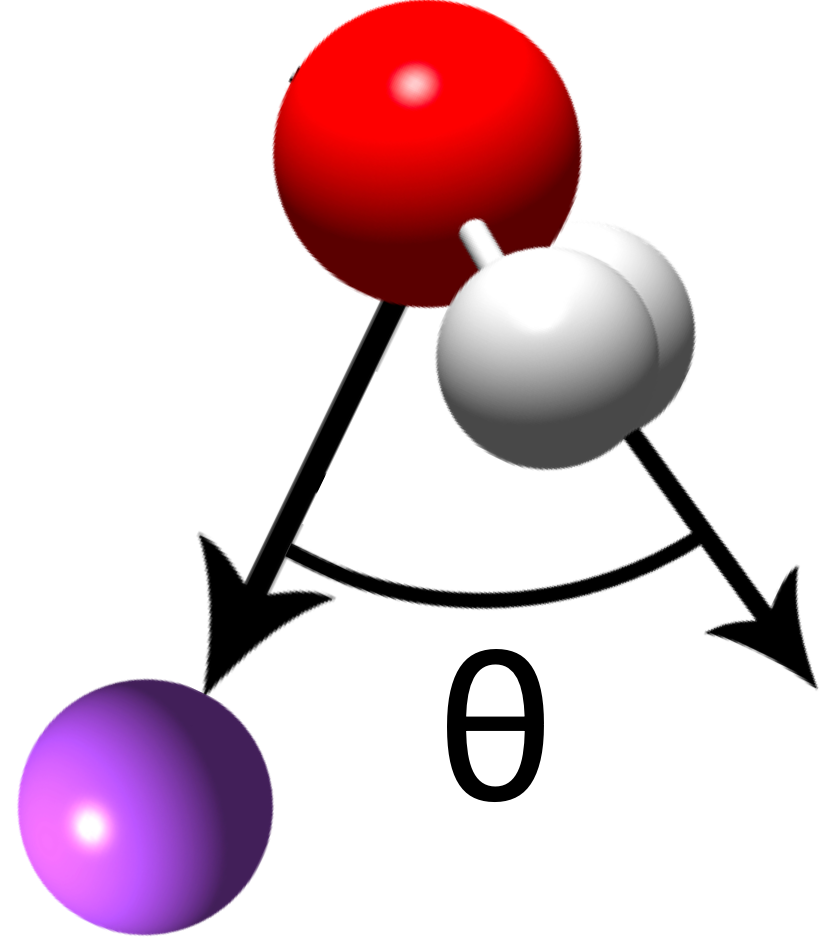

Supplement: Supplementary file 6 — Source Data [file 41467_2024_46772_MOESM6_ESM.zip › fig_data/fig1/theta.png]

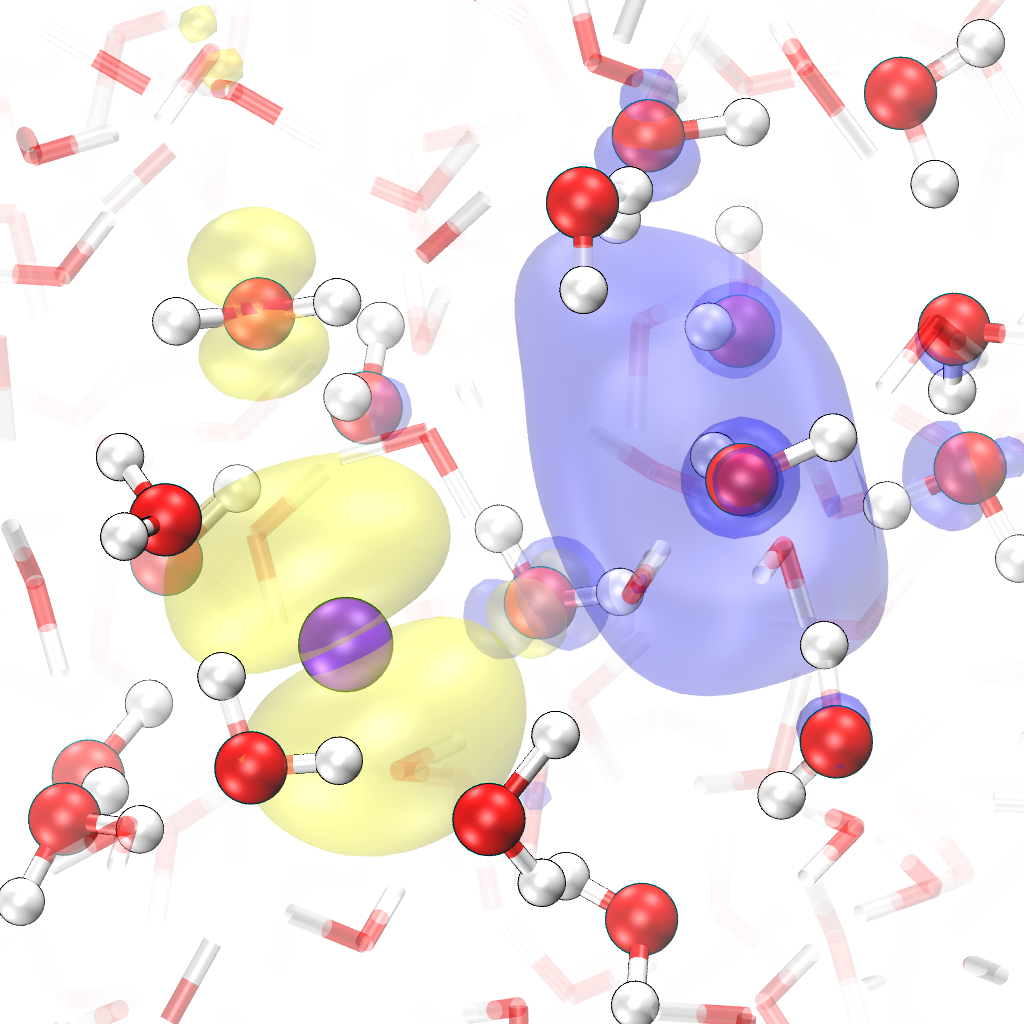

Supplement: Supplementary file 6 — Source Data [file 41467_2024_46772_MOESM6_ESM.zip › fig_data/fig2/ss.png]

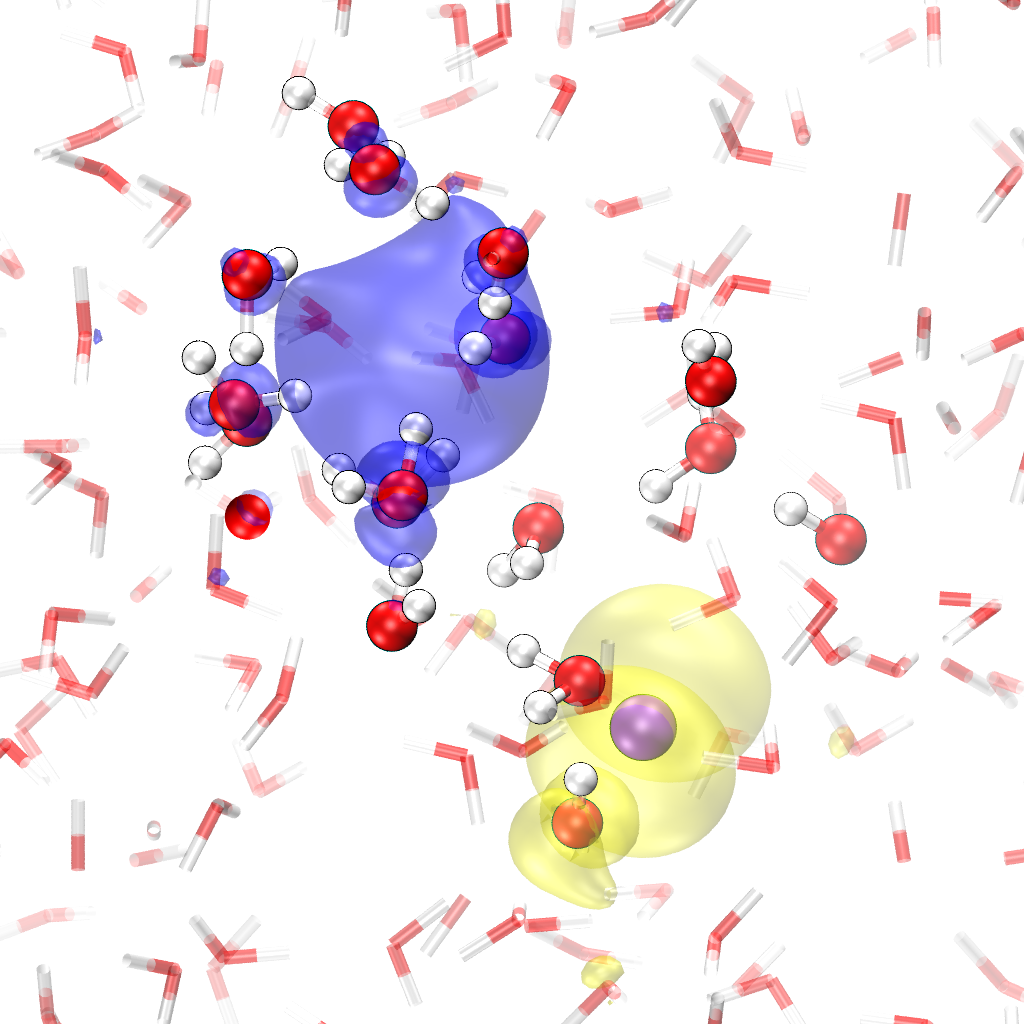

Supplement: Supplementary file 6 — Source Data [file 41467_2024_46772_MOESM6_ESM.zip › fig_data/fig2/he.png]

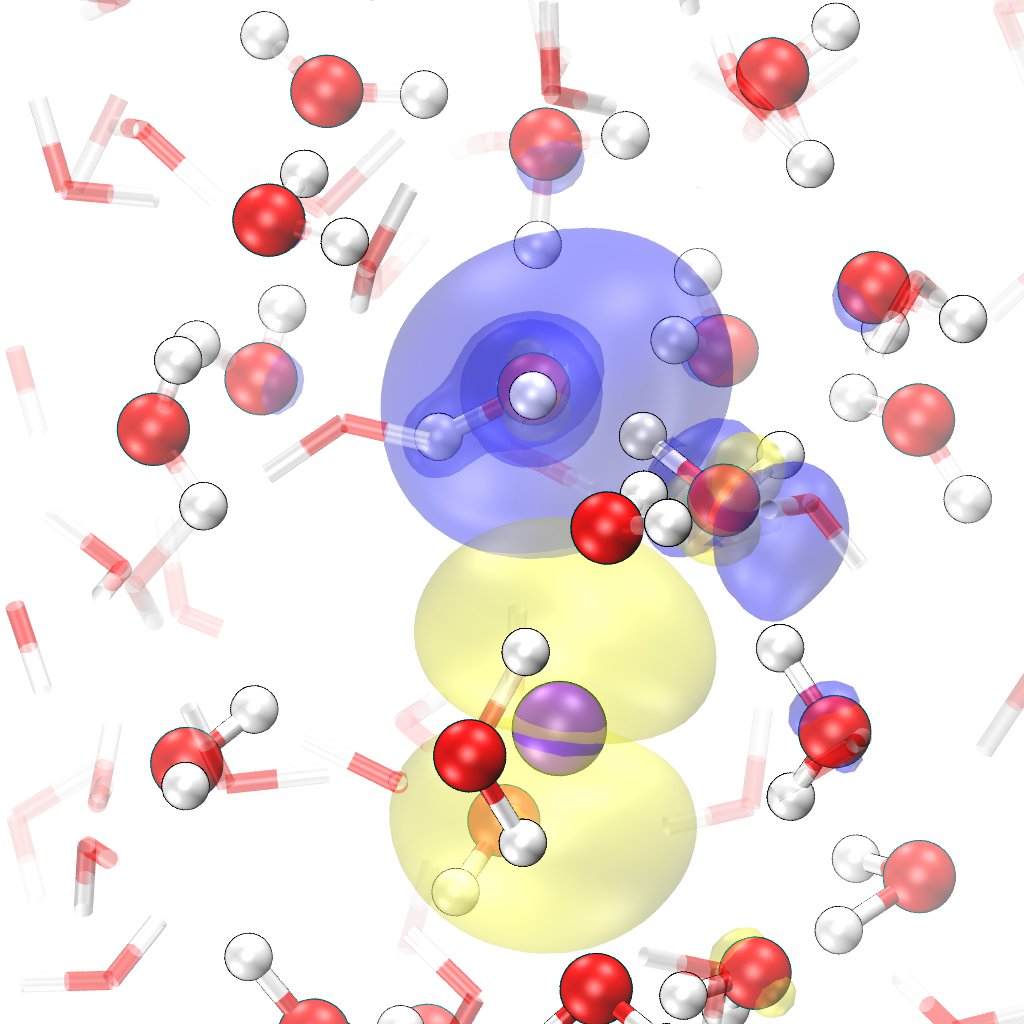

Supplement: Supplementary file 6 — Source Data [file 41467_2024_46772_MOESM6_ESM.zip › fig_data/fig2/share.png]

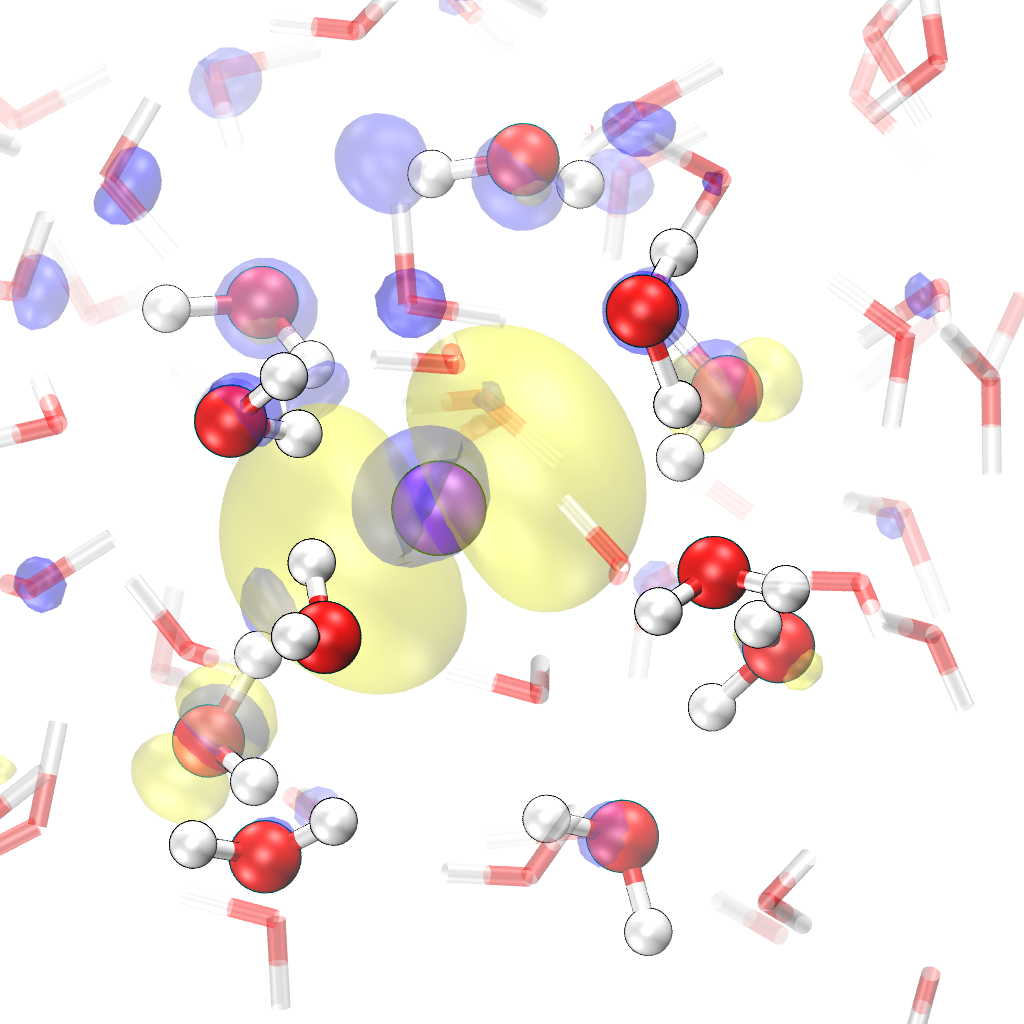

Supplement: Supplementary file 6 — Source Data [file 41467_2024_46772_MOESM6_ESM.zip › fig_data/fig2/ctts.png]

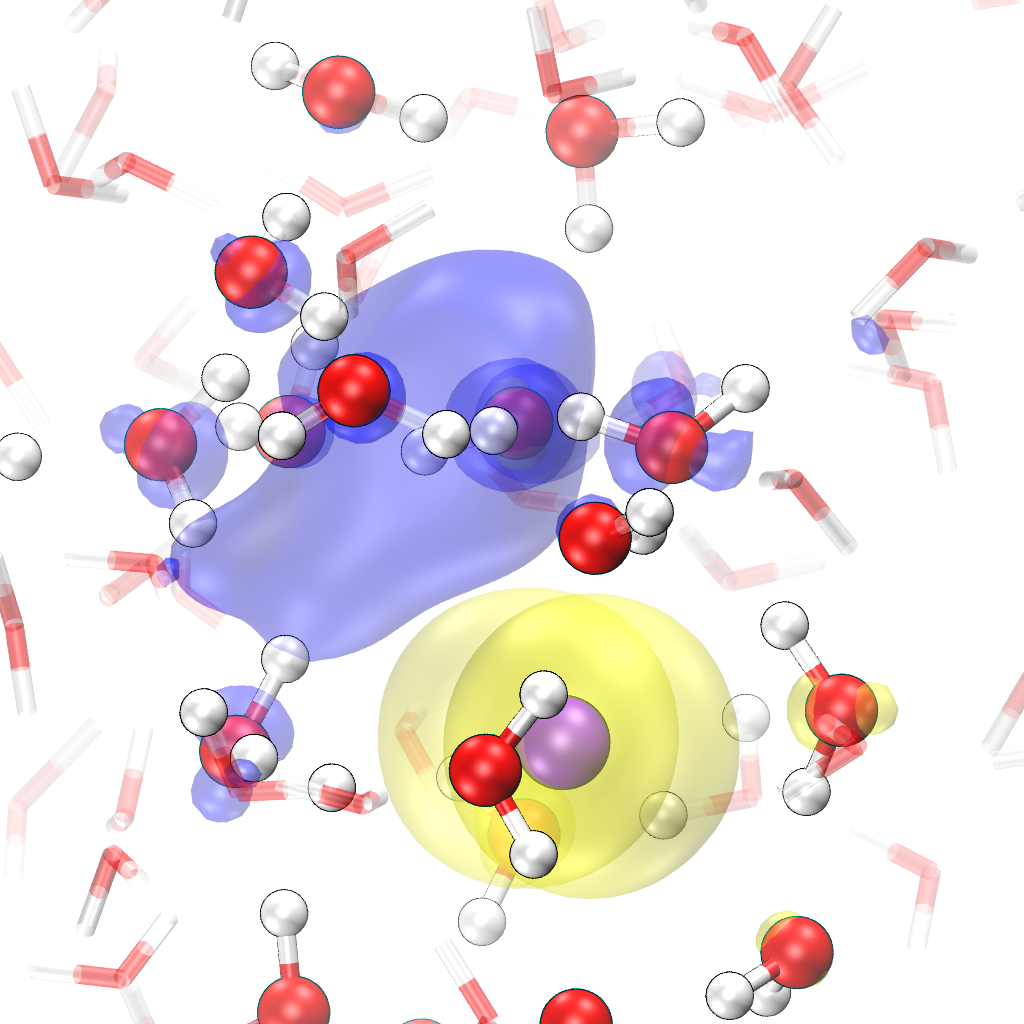

Supplement: Supplementary file 6 — Source Data [file 41467_2024_46772_MOESM6_ESM.zip › fig_data/fig2/cp.png]

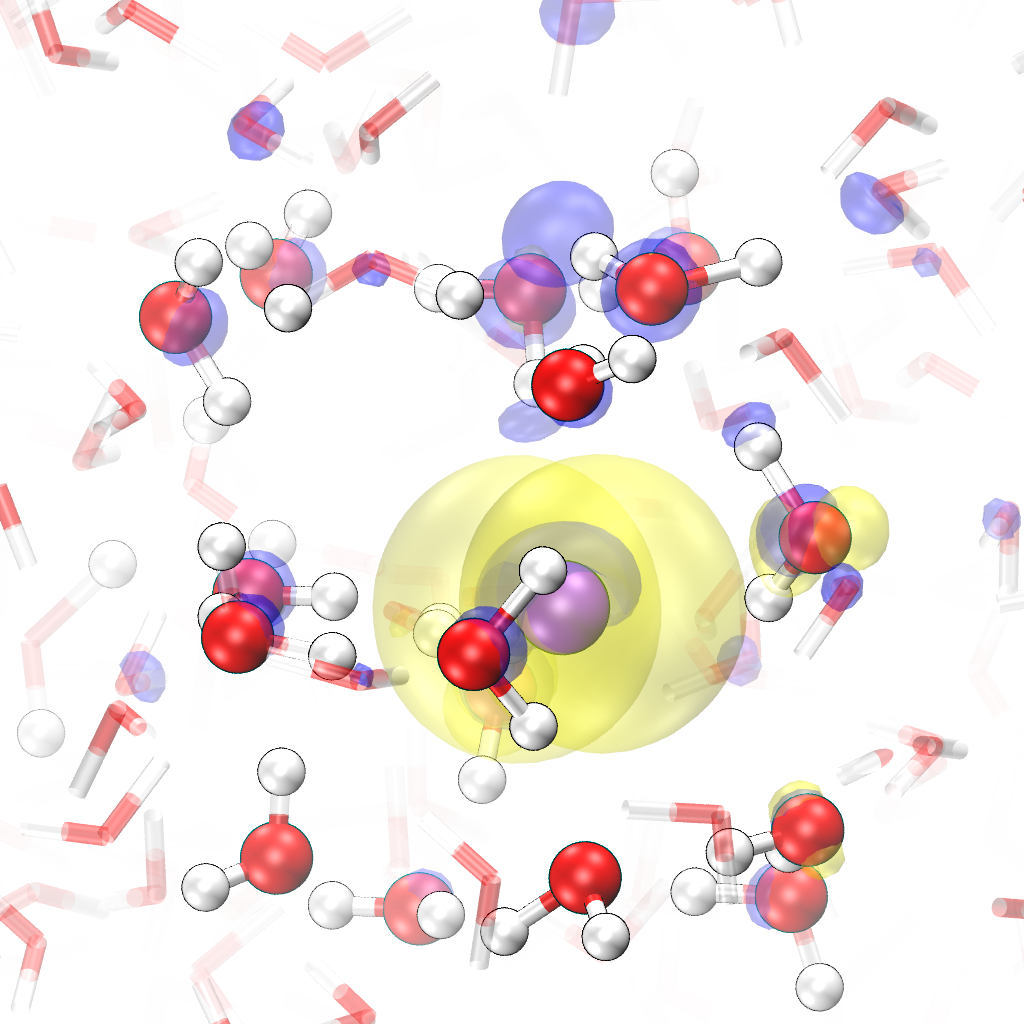

Supplement: Supplementary file 6 — Source Data [file 41467_2024_46772_MOESM6_ESM.zip › fig_data/fig2/ctts2.png]
